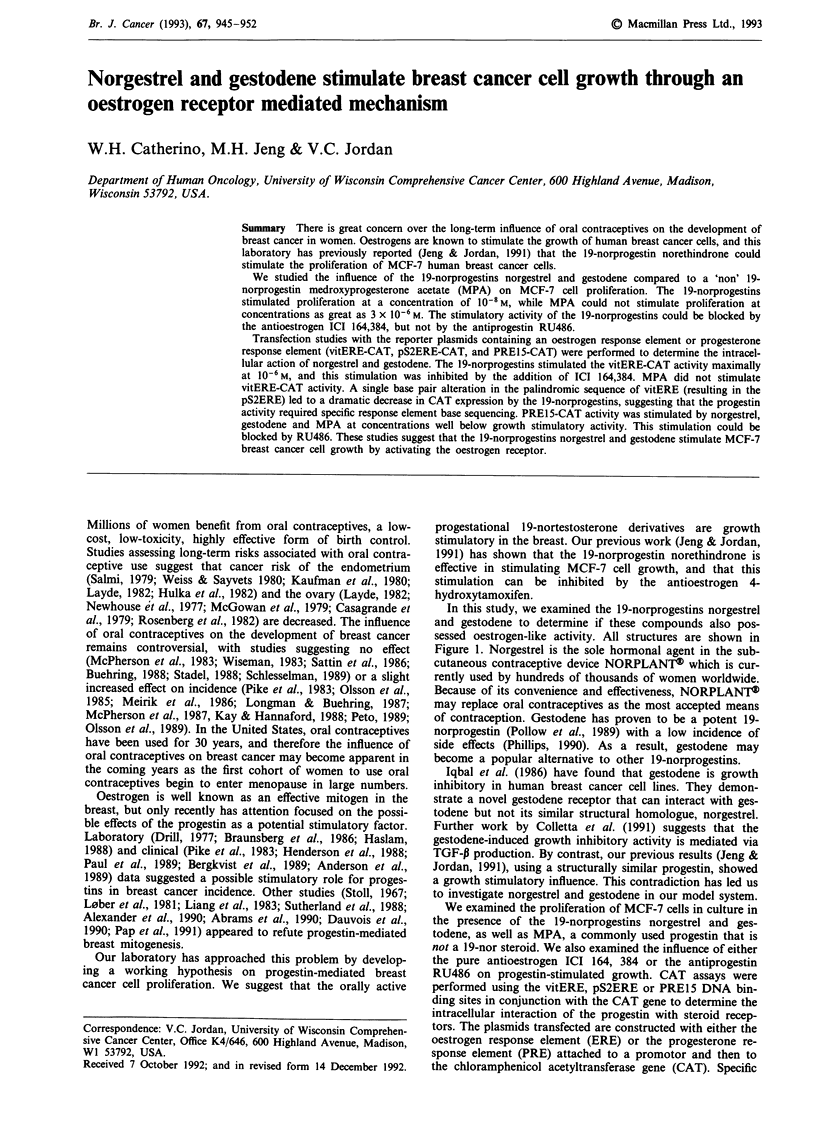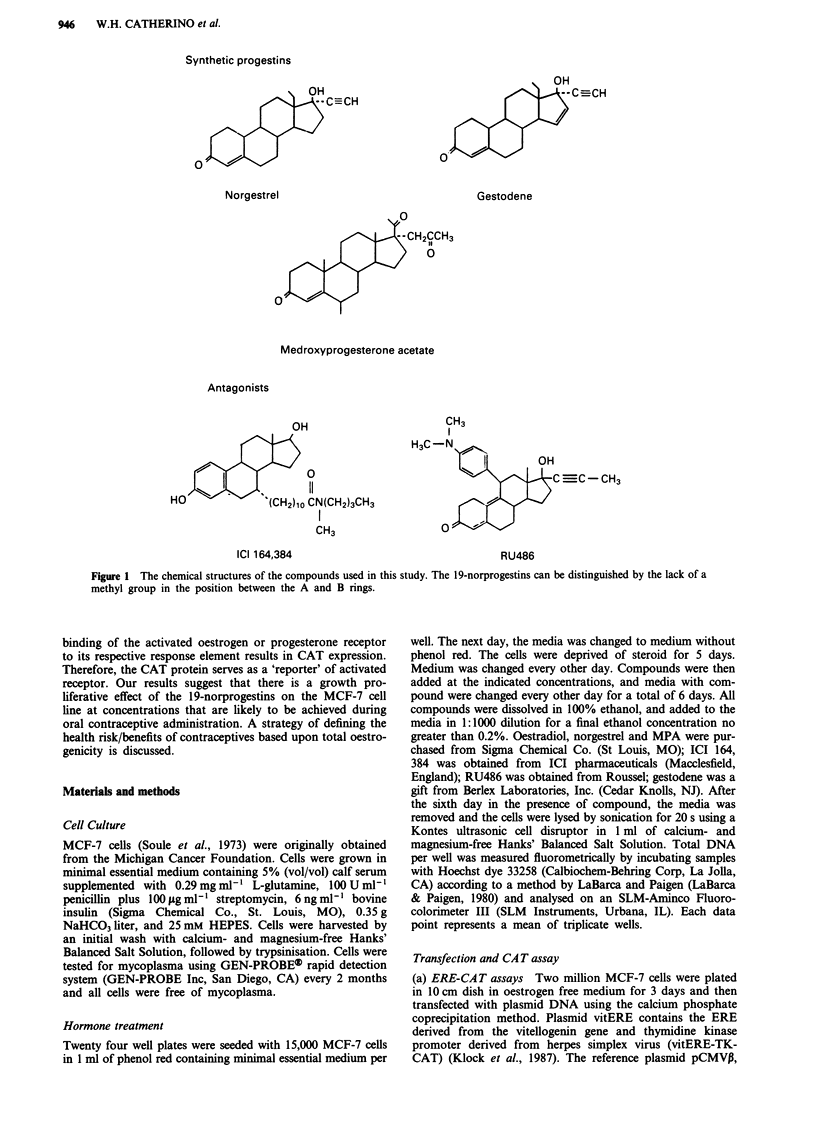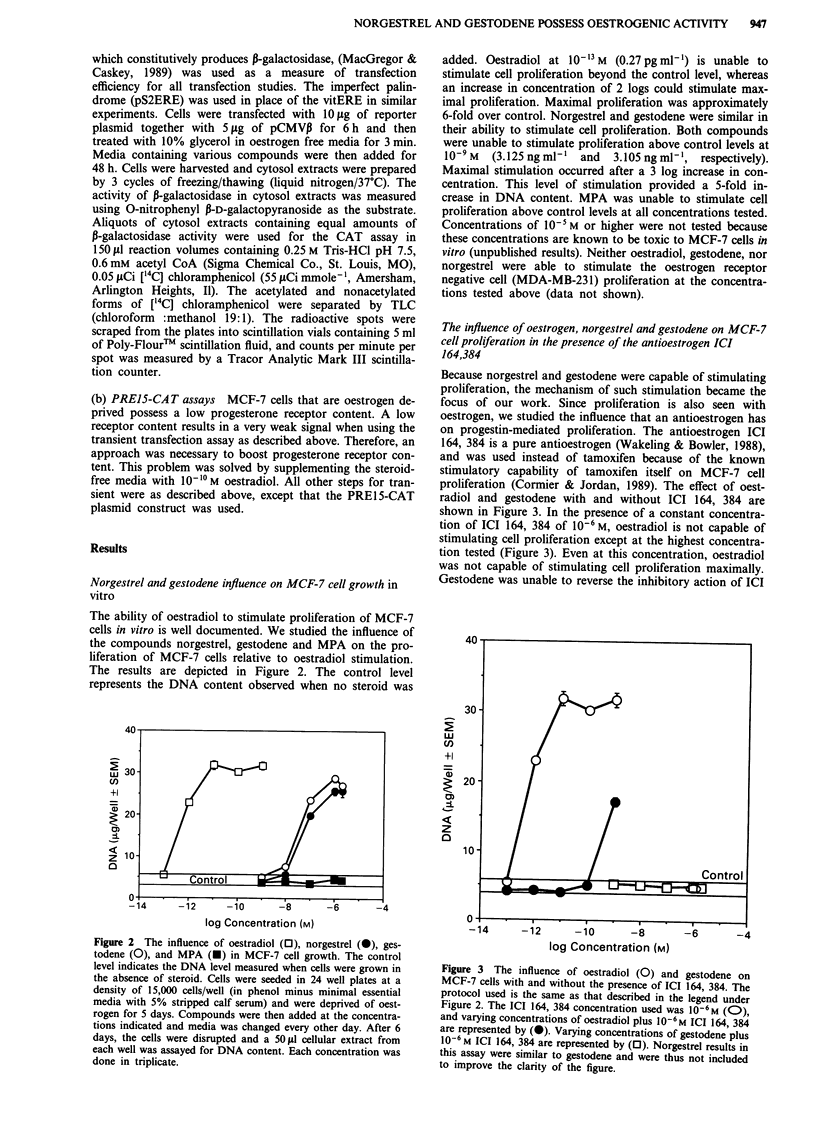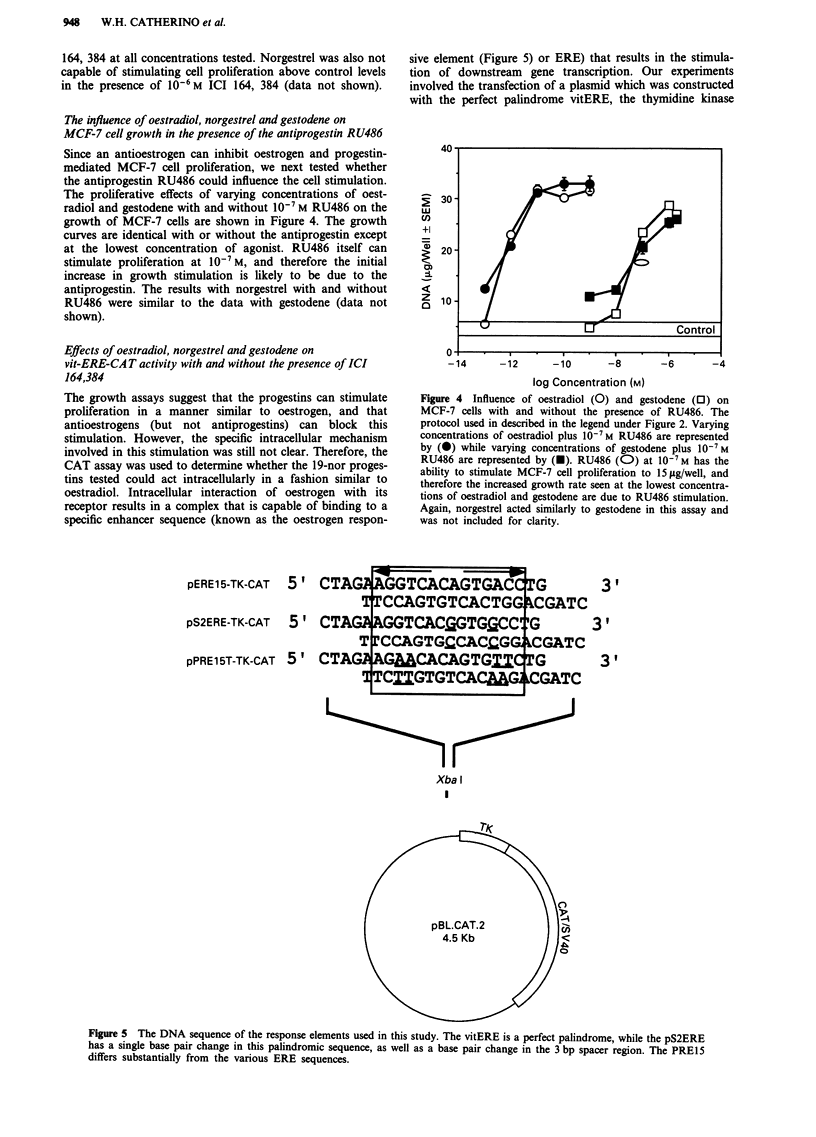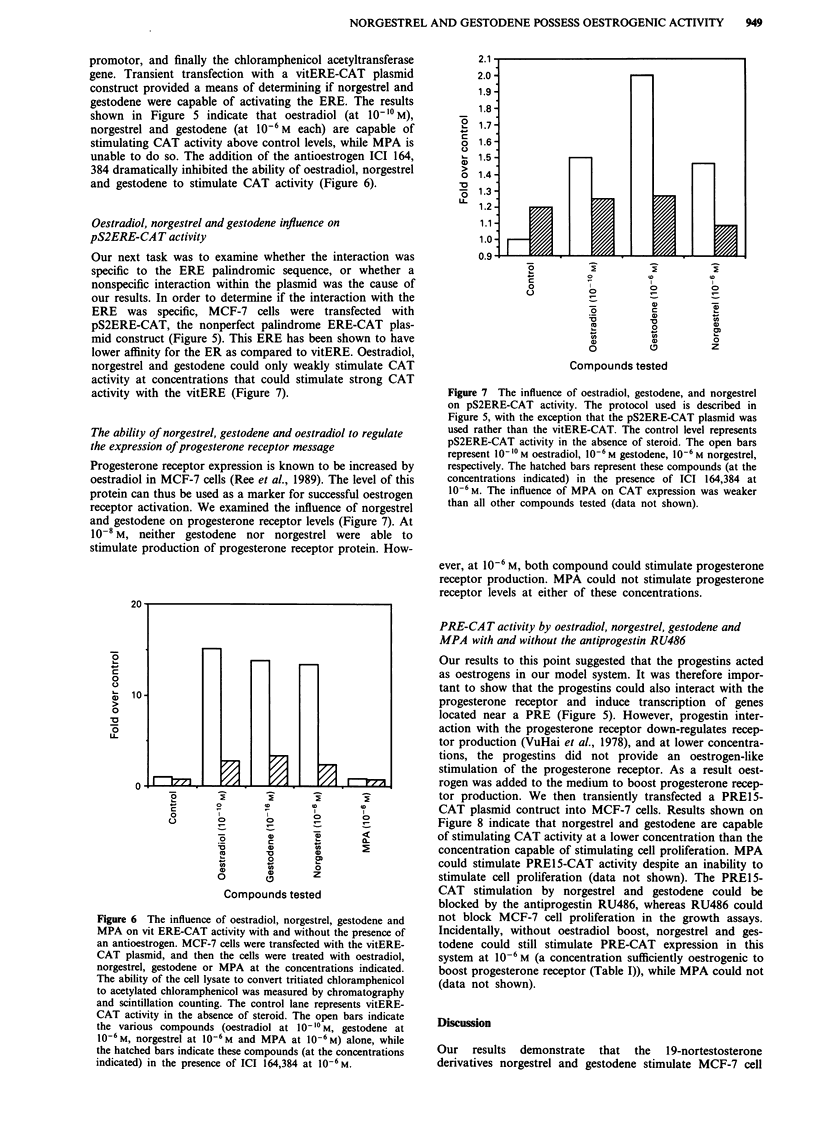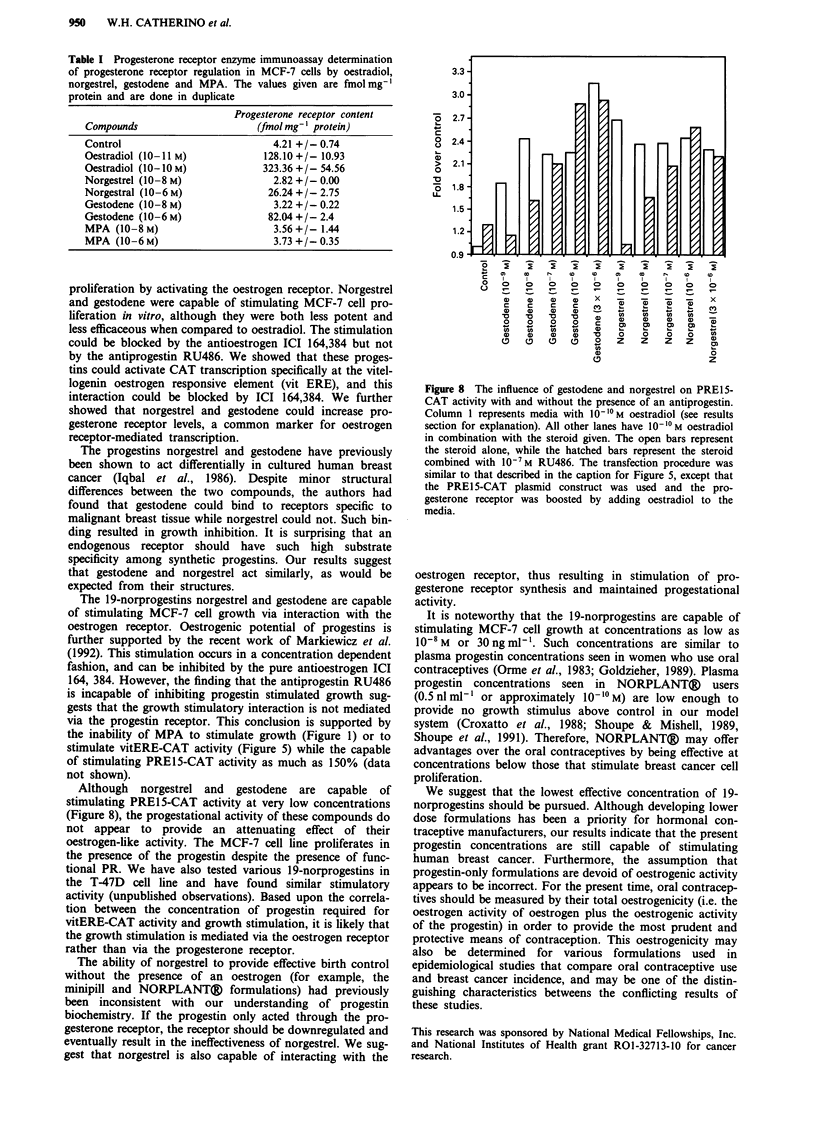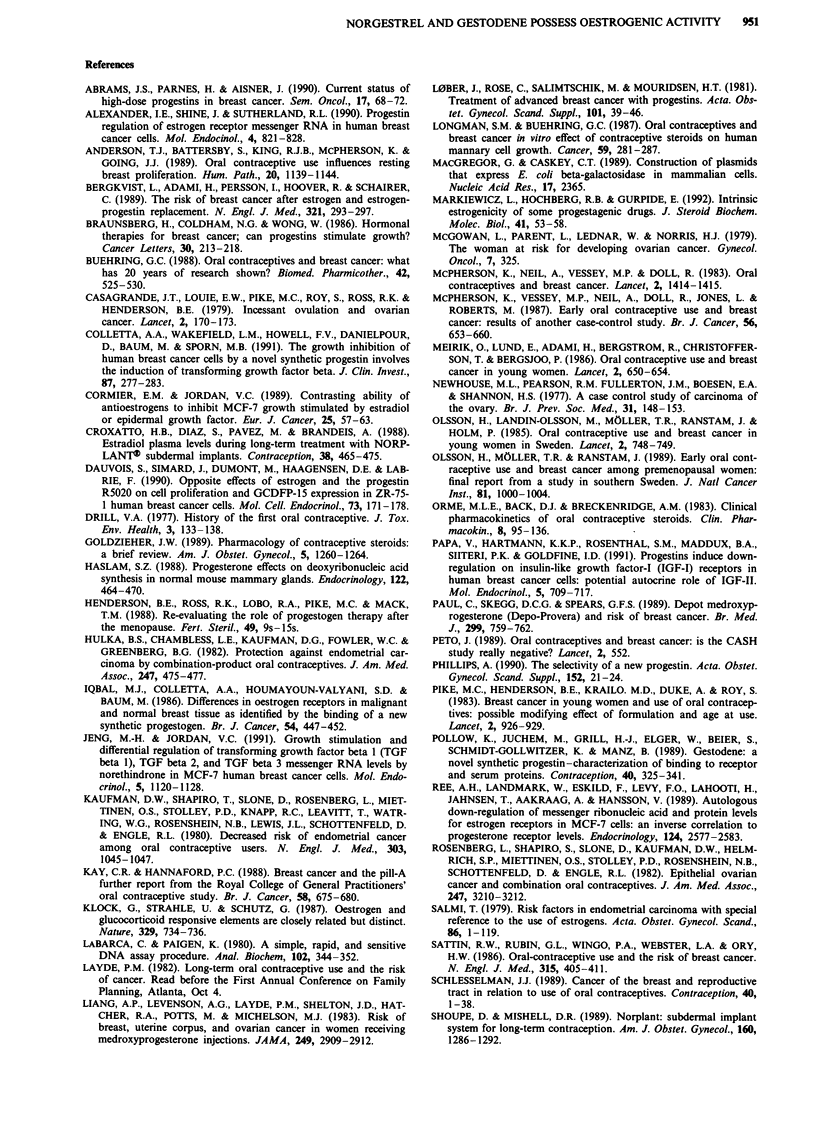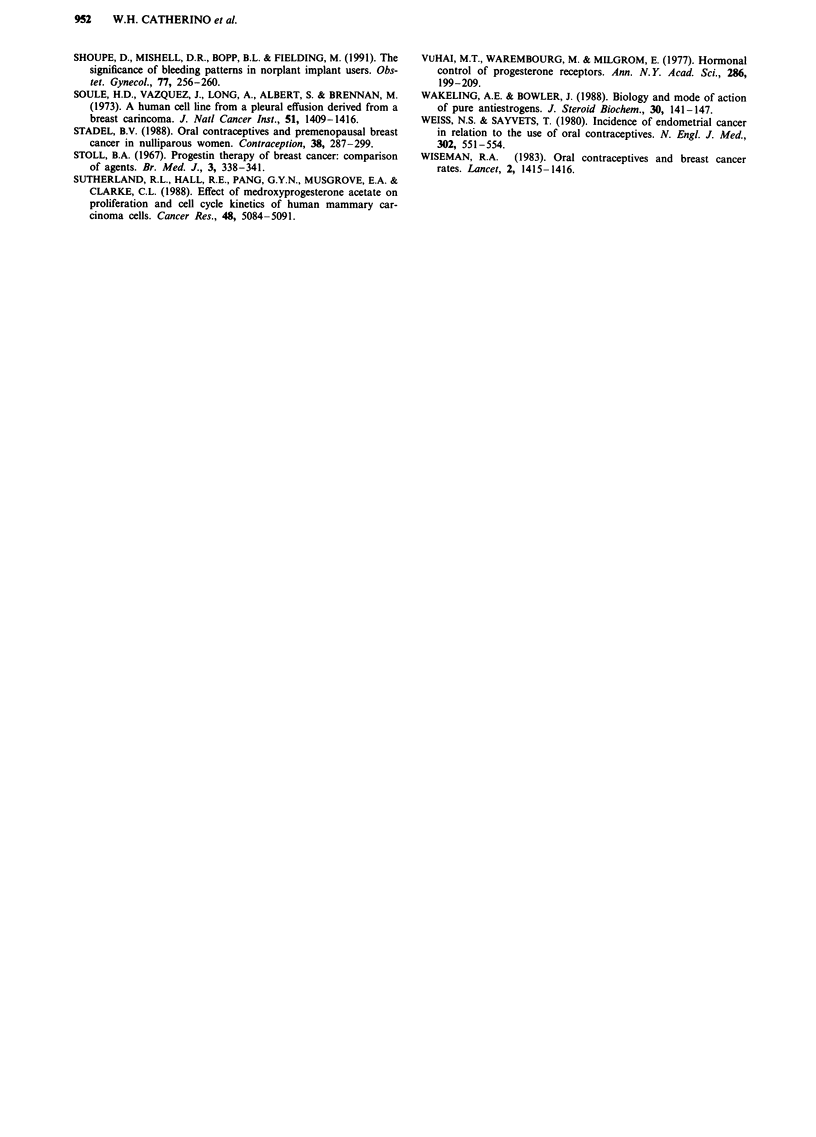# Norgestrel and gestodene stimulate breast cancer cell growth through an oestrogen receptor mediated mechanism.

**DOI:** 10.1038/bjc.1993.175

**Published:** 1993-05

**Authors:** W. H. Catherino, M. H. Jeng, V. C. Jordan

**Affiliations:** Department of Human Oncology, University of Wisconsin Comprehensive Cancer Center, Madison 53792.

## Abstract

There is great concern over the long-term influence of oral contraceptives on the development of breast cancer in women. Oestrogens are known to stimulate the growth of human breast cancer cells, and this laboratory has previously reported (Jeng & Jordan, 1991) that the 19-norprogestin norethindrone could stimulate the proliferation of MCF-7 human breast cancer cells. We studied the influence of the 19-norprogestins norgestrel and gestodene compared to a 'non' 19-norprogestin medroxyprogesterone acetate (MPA) on MCF-7 cell proliferation. The 19-norprogestins stimulated proliferation at a concentration of 10(-8) M, while MPA could not stimulate proliferation at concentrations as great as 3 x 10(-6) M. The stimulatory activity of the 19-norprogestins could be blocked by the antioestrogen ICI 164,384, but not by the antiprogestin RU486. Transfection studies with the reporter plasmids containing an oestrogen response element or progesterone response element (vitERE-CAT, pS2ERE-CAT, and PRE15-CAT) were performed to determine the intracellular action of norgestrel and gestodene. The 19-norprogestins stimulated the vitERE-CAT activity maximally at 10(-6) M, and this stimulation was inhibited by the addition of ICI 164,384. MPA did not stimulate vitERE-CAT activity. A single base pair alteration in the palindromic sequence of vitERE (resulting in the pS2ERE) led to a dramatic decrease in CAT expression by the 19-norprogestins, suggesting that the progestin activity required specific response element base sequencing. PRE15-CAT activity was stimulated by norgestrel, gestodene and MPA at concentrations well below growth stimulatory activity. This stimulation could be blocked by RU486. These studies suggest that the 19-norprogestins norgestrel and gestodene stimulate MCF-7 breast cancer cell growth by activating the oestrogen receptor.


Br. J. Cancer (1993), 67, 945-952 © Macmillan Press Ltd., 1993

### Norgestrel and gestodene stimulate breast cancer cell growth through an oestrogen receptor mediated mechanism

W.H. Catherino, M.H. Jeng & V.C. Jordan

*Department of Human Oncology, University of Wisconsin Comprehensive Cancer Center, 600 Highland Avenue, Madison, Wisconsin 53792, USA.*

**Summary** There is great concern over the long-term influence of oral contraceptives on the development of breast cancer in women. Oestrogens are known to stimulate the growth of human breast cancer cells, and this laboratory has previously reported (Jeng & Jordan, 1991) that the 19-norprogestin norethindrone could stimulate the proliferation of MCF-7 human breast cancer cells.

We studied the influence of the 19-norprogestins norgestrel and gestodene compared to a 'non' 19-norprogestin medroxyprogesterone acetate (MPA) on MCF-7 cell proliferation. The 19-norprogestins stimulated proliferation at a concentration of $10^{-8}$ M, while MPA could not stimulate proliferation at concentrations as great as $3 \times 10^{-6}$ M. The stimulatory activity of the 19-norprogestins could be blocked by the antioestrogen ICI 164,384, but not by the antiprogestin RU486.

Transfection studies with the reporter plasmids containing an oestrogen response element or progesterone response element (vitERE-CAT, pS2ERE-CAT, and PRE15-CAT) were performed to determine the intracellular action of norgestrel and gestodene. The 19-norprogestins stimulated the vitERE-CAT activity maximally at $10^{-6}$ M, and this stimulation was inhibited by the addition of ICI 164,384. MPA did not stimulate vitERE-CAT activity. A single base pair alteration in the palindromic sequence of vitERE (resulting in the pS2ERE) led to a dramatic decrease in CAT expression by the 19-norprogestins, suggesting that the progestin activity required specific response element base sequencing. PRE15-CAT activity was stimulated by norgestrel, gestodene and MPA at concentrations well below growth stimulatory activity. This stimulation could be blocked by RU486. These studies suggest that the 19-norprogestins norgestrel and gestodene stimulate MCF-7 breast cancer cell growth by activating the oestrogen receptor.

Millions of women benefit from oral contraceptives, a low-cost, low-toxicity, highly effective form of birth control. Studies assessing long-term risks associated with oral contraceptive use suggest that cancer risk of the endometrium (Salmi, 1979; Weiss & Sayvets 1980; Kaufman *et al.*, 1980; Layde, 1982; Hulka *et al.*, 1982) and the ovary (Layde, 1982; Newhouse *et al.*, 1977; McGowan *et al.*, 1979; Casagrande *et al.*, 1979; Rosenberg *et al.*, 1982) are decreased. The influence of oral contraceptives on the development of breast cancer remains controversial, with studies suggesting no effect (McPherson *et al.*, 1983; Wiseman, 1983; Sattin *et al.*, 1986; Buehring, 1988; Stadel, 1988; Schlesselman, 1989) or a slight increased effect on incidence (Pike *et al.*, 1983; Olsson *et al.*, 1985; Meirik *et al.*, 1986; Longman & Buehring, 1987; McPherson *et al.*, 1987, Kay & Hannaford, 1988; Peto, 1989; Olsson *et al.*, 1989). In the United States, oral contraceptives have been used for 30 years, and therefore the influence of oral contraceptives on breast cancer may become apparent in the coming years as the first cohort of women to use oral contraceptives begin to enter menopause in large numbers.

Oestrogen is well known as an effective mitogen in the breast, but only recently has attention focused on the possible effects of the progestin as a potential stimulatory factor. Laboratory (Drill, 1977; Braunsberg *et al.*, 1986; Haslam, 1988) and clinical (Pike *et al.*, 1983; Henderson *et al.*, 1988; Paul *et al.*, 1989; Bergkvist *et al.*, 1989; Anderson *et al.*, 1989) data suggested a possible stimulatory role for progestins in breast cancer incidence. Other studies (Stoll, 1967; Løber *et al.*, 1981; Liang *et al.*, 1983; Sutherland *et al.*, 1988; Alexander *et al.*, 1990; Abrams *et al.*, 1990; Dauvois *et al.*, 1990; Pap *et al.*, 1991) appeared to refute progestin-mediated breast mitogenesis.

Our laboratory has approached this problem by developing a working hypothesis on progestin-mediated breast cancer cell proliferation. We suggest that the orally active progestational 19-nortestosterone derivatives are growth stimulatory in the breast. Our previous work (Jeng & Jordan, 1991) has shown that the 19-norprogestin norethindrone is effective in stimulating MCF-7 cell growth, and that this stimulation can be inhibited by the antioestrogen 4-hydroxytamoxifen.

In this study, we examined the 19-norprogestins norgestrel and gestodene to determine if these compounds also possessed oestrogen-like activity. All structures are shown in Figure 1. Norgestrel is the sole hormonal agent in the subcutaneous contraceptive device NORPLANT® which is currently used by hundreds of thousands of women worldwide. Because of its convenience and effectiveness, NORPLANT® may replace oral contraceptives as the most accepted means of contraception. Gestodene has proven to be a potent 19-norprogestin (Pollow *et al.*, 1989) with a low incidence of side effects (Phillips, 1990). As a result, gestodene may become a popular alternative to other 19-norprogestins.

Iqbal *et al.* (1986) have found that gestodene is growth inhibitory in human breast cancer cell lines. They demonstrate a novel gestodene receptor that can interact with gestodene but not its similar structural homologue, norgestrel. Further work by Colletta *et al.* (1991) suggests that the gestodene-induced growth inhibitory activity is mediated via TGF-$\beta$ production. By contrast, our previous results (Jeng & Jordan, 1991), using a structurally similar progestin, showed a growth stimulatory influence. This contradiction has led us to investigate norgestrel and gestodene in our model system.

We examined the proliferation of MCF-7 cells in culture in the presence of the 19-norprogestins norgestrel and gestodene, as well as MPA, a commonly used progestin that is *not* a 19-nor steroid. We also examined the influence of either the pure antioestrogen ICI 164, 384 or the antiprogestin RU486 on progestin-stimulated growth. CAT assays were performed using the vitERE, pS2ERE or PRE15 DNA binding sites in conjunction with the CAT gene to determine the intracellular interaction of the progestin with steroid receptors. The plasmids transfected are constructed with either the oestrogen response element (ERE) or the progesterone response element (PRE) attached to a promotor and then to the chloramphenicol acetyltransferase gene (CAT). Specific

Correspondence: V.C. Jordan, University of Wisconsin Comprehensive Cancer Center, Office K4/646, 600 Highland Avenue, Madison, WI 53792, USA.
Received 7 October 1992; and in revised form 14 December 1992.

**Figure 1** The chemical structures of the compounds used in this study. The 19-norprogestins can be distinguished by the lack of a methyl group in the position between the A and B rings.

binding of the activated oestrogen or progesterone receptor to its respective response element results in CAT expression. Therefore, the CAT protein serves as a 'reporter' of activated receptor. Our results suggest that there is a growth proliferative effect of the 19-norprogestins on the MCF-7 cell line at concentrations that are likely to be achieved during oral contraceptive administration. A strategy of defining the health risk/benefits of contraceptives based upon total oestrogenicity is discussed.

### Materials and methods

### *Cell Culture*

MCF-7 cells (Soule *et al.*, 1973) were originally obtained from the Michigan Cancer Foundation. Cells were grown in minimal essential medium containing 5% (vol/vol) calf serum supplemented with 0.29 mg $ml^{-1}$ L-glutamine, 100 U $ml^{-1}$ penicillin plus 100 $\mu g$ $ml^{-1}$ streptomycin, 6 ng $ml^{-1}$ bovine insulin (Sigma Chemical Co., St. Louis, MO), 0.35 g $NaHCO_3$ liter, and 25 mM HEPES. Cells were harvested by an initial wash with calcium- and magnesium-free Hanks' Balanced Salt Solution, followed by trypsinisation. Cells were tested for mycoplasma using GEN-PROBE® rapid detection system (GEN-PROBE Inc, San Diego, CA) every 2 months and all cells were free of mycoplasma.

### *Hormone treatment*

Twenty four well plates were seeded with 15,000 MCF-7 cells in 1 ml of phenol red containing minimal essential medium per well. The next day, the media was changed to medium without phenol red. The cells were deprived of steroid for 5 days. Medium was changed every other day. Compounds were then added at the indicated concentrations, and media with compound were changed every other day for a total of 6 days. All compounds were dissolved in 100% ethanol, and added to the media in 1:1000 dilution for a final ethanol concentration no greater than 0.2%. Oestradiol, norgestrel and MPA were purchased from Sigma Chemical Co. (St Louis, MO); ICI 164, 384 was obtained from ICI pharmaceuticals (Macclesfield, England); RU486 was obtained from Roussel; gestodene was a gift from Berlex Laboratories, Inc. (Cedar Knolls, NJ). After the sixth day in the presence of compound, the media was removed and the cells were lysed by sonication for 20 s using a Kontes ultrasonic cell disruptor in 1 ml of calcium- and magnesium-free Hanks' Balanced Salt Solution. Total DNA per well was measured fluorometrically by incubating samples with Hoechst dye 33258 (Calbiochem-Behring Corp, La Jolla, CA) according to a method by LaBarca and Paigen (LaBarca & Paigen, 1980) and analysed on an SLM-Aminco Fluorocolorimeter III (SLM Instruments, Urbana, IL). Each data point represents a mean of triplicate wells.

### *Transfection and CAT assay*

(a) *ERE-CAT assays* Two million MCF-7 cells were plated in 10 cm dish in oestrogen free medium for 3 days and then transfected with plasmid DNA using the calcium phosphate coprecipitation method. Plasmid vitERE contains the ERE derived from the vitellogenin gene and thymidine kinase promoter derived from herpes simplex virus (vitERE-TK-CAT) (Klock *et al.*, 1987). The reference plasmid pCMV$\beta$, which constitutively produces β-galactosidase, (MacGregor & Caskey, 1989) was used as a measure of transfection efficiency for all transfection studies. The imperfect palindrome (pS2ERE) was used in place of the vitERE in similar experiments. Cells were transfected with 10 μg of reporter plasmid together with 5 μg of pCMVβ for 6 h and then treated with 10% glycerol in oestrogen free media for 3 min. Media containing various compounds were then added for 48 h. Cells were harvested and cytosol extracts were prepared by 3 cycles of freezing/thawing (liquid nitrogen/37°C). The activity of β-galactosidase in cytosol extracts was measured using O-nitrophenyl β-D-galactopyranoside as the substrate. Aliquots of cytosol extracts containing equal amounts of β-galactosidase activity were used for the CAT assay in 150 μl reaction volumes containing 0.25 M Tris-HCl pH 7.5, 0.6 mM acetyl CoA (Sigma Chemical Co., St. Louis, MO), 0.05 μCi [$^{14}$C] chloramphenicol (55 μCi mmole$^{-1}$, Amersham, Arlington Heights, Il). The acetylated and nonacetylated forms of [$^{14}$C] chloramphenicol were separated by TLC (chloroform :methanol 19:1). The radioactive spots were scraped from the plates into scintillation vials containing 5 ml of Poly-Flour™ scintillation fluid, and counts per minute per spot was measured by a Tracor Analytic Mark III scintillation counter.

(b) *PRE15-CAT assays* MCF-7 cells that are oestrogen deprived possess a low progesterone receptor content. A low receptor content results in a very weak signal when using the transient transfection assay as described above. Therefore, an approach was necessary to boost progesterone receptor content. This problem was solved by supplementing the steroid-free media with $10^{-10}$ M oestradiol. All other steps for transient were as described above, except that the PRE15-CAT plasmid construct was used.

### Results

*Norgestrel and gestodene influence on MCF-7 cell growth* in vitro

The ability of oestradiol to stimulate proliferation of MCF-7 cells *in vitro* is well documented. We studied the influence of the compounds norgestrel, gestodene and MPA on the proliferation of MCF-7 cells relative to oestradiol stimulation. The results are depicted in Figure 2. The control level represents the DNA content observed when no steroid was added. Oestradiol at $10^{-13}$ M (0.27 pg ml$^{-1}$) is unable to stimulate cell proliferation beyond the control level, whereas an increase in concentration of 2 logs could stimulate maximal proliferation. Maximal proliferation was approximately 6-fold over control. Norgestrel and gestodene were similar in their ability to stimulate cell proliferation. Both compounds were unable to stimulate proliferation above control levels at $10^{-9}$ M (3.125 ng ml$^{-1}$ and 3.105 ng ml$^{-1}$, respectively). Maximal stimulation occurred after a 3 log increase in concentration. This level of stimulation provided a 5-fold increase in DNA content. MPA was unable to stimulate cell proliferation above control levels at all concentrations tested. Concentrations of $10^{-5}$ M or higher were not tested because these concentrations are known to be toxic to MCF-7 cells *in vitro* (unpublished results). Neither oestradiol, gestodene, nor norgestrel were able to stimulate the oestrogen receptor negative cell (MDA-MB-231) proliferation at the concentrations tested above (data not shown).

**Figure 2** The influence of oestradiol (□), norgestrel (●), gestodene (○), and MPA (■) in MCF-7 cell growth. The control level indicates the DNA level measured when cells were grown in the absence of steroid. Cells were seeded in 24 well plates at a density of 15,000 cells/well (in phenol minus minimal essential media with 5% stripped calf serum) and were deprived of oestrogen for 5 days. Compounds were then added at the concentrations indicated and media was changed every other day. After 6 days, the cells were disrupted and a 50 μl cellular extract from each well was assayed for DNA content. Each concentration was done in triplicate.

*The influence of oestrogen, norgestrel and gestodene on MCF-7 cell proliferation in the presence of the antioestrogen ICI 164,384*

Because norgestrel and gestodene were capable of stimulating proliferation, the mechanism of such stimulation became the focus of our work. Since proliferation is also seen with oestrogen, we studied the influence that an antioestrogen has on progestin-mediated proliferation. The antioestrogen ICI 164, 384 is a pure antioestrogen (Wakeling & Bowler, 1988), and was used instead of tamoxifen because of the known stimulatory capability of tamoxifen itself on MCF-7 cell proliferation (Cormier & Jordan, 1989). The effect of oestradiol and gestodene with and without ICI 164, 384 are shown in Figure 3. In the presence of a constant concentration of ICI 164, 384 of $10^{-6}$ M, oestradiol is not capable of stimulating cell proliferation except at the highest concentration tested (Figure 3). Even at this concentration, oestradiol was not capable of stimulating cell proliferation maximally. Gestodene was unable to reverse the inhibitory action of ICI

**Figure 3** The influence of oestradiol (○) and gestodene on MCF-7 cells with and without the presence of ICI 164, 384. The protocol used is the same as that described in the legend under Figure 2. The ICI 164, 384 concentration used was $10^{-6}$ M (○), and varying concentrations of oestradiol plus $10^{-6}$ M ICI 164, 384 are represented by (●). Varying concentrations of gestodene plus $10^{-6}$ M ICI 164, 384 are represented by (□). Norgestrel results in this assay were similar to gestodene and were thus not included to improve the clarity of the figure.

164, 384 at all concentrations tested. Norgestrel was also not capable of stimulating cell proliferation above control levels in the presence of $10^{-6}$ M ICI 164, 384 (data not shown).

*The influence of oestradiol, norgestrel and gestodene on MCF-7 cell growth in the presence of the antiprogestin RU486*

Since an antioestrogen can inhibit oestrogen and progestin-mediated MCF-7 cell proliferation, we next tested whether the antiprogestin RU486 could influence the cell stimulation. The proliferative effects of varying concentrations of oestradiol and gestodene with and without $10^{-7}$ M RU486 on the growth of MCF-7 cells are shown in Figure 4. The growth curves are identical with or without the antiprogestin except at the lowest concentration of agonist. RU486 itself can stimulate proliferation at $10^{-7}$ M, and therefore the initial increase in growth stimulation is likely to be due to the antiprogestin. The results with norgestrel with and without RU486 were similar to the data with gestodene (data not shown).

*Effects of oestradiol, norgestrel and gestodene on vit-ERE-CAT activity with and without the presence of ICI 164,384*

The growth assays suggest that the progestins can stimulate proliferation in a manner similar to oestrogen, and that antioestrogens (but not antiprogestins) can block this stimulation. However, the specific intracellular mechanism involved in this stimulation was still not clear. Therefore, the CAT assay was used to determine whether the 19-nor progestins tested could act intracellularly in a fashion similar to oestradiol. Intracellular interaction of oestrogen with its receptor results in a complex that is capable of binding to a specific enhancer sequence (known as the oestrogen responsive element (Figure 5) or ERE) that results in the stimulation of downstream gene transcription. Our experiments involved the transfection of a plasmid which was constructed with the perfect palindrome vitERE, the thymidine kinase

**Figure 4** Influence of oestradiol (○) and gestodene (□) on MCF-7 cells with and without the presence of RU486. The protocol used in described in the legend under Figure 2. Varying concentrations of oestradiol plus $10^{-7}$ M RU486 are represented by (●) while varying concentrations of gestodene plus $10^{-7}$ M RU486 are represented by (■). RU486 (⬭) at $10^{-7}$ M has the ability to stimulate MCF-7 cell proliferation to 15 µg/well, and therefore the increased growth rate seen at the lowest concentrations of oestradiol and gestodene are due to RU486 stimulation. Again, norgestrel acted similarly to gestodene in this assay and was not included for clarity.

**Figure 5** The DNA sequence of the response elements used in this study. The vitERE is a perfect palindrome, while the pS2ERE has a single base pair change in this palindromic sequence, as well as a base pair change in the 3 bp spacer region. The PRE15 differs substantially from the various ERE sequences.

promotor, and finally the chloramphenicol acetyltransferase gene. Transient transfection with a vitERE-CAT plasmid construct provided a means of determining if norgestrel and gestodene were capable of activating the ERE. The results shown in Figure 5 indicate that oestradiol (at $10^{-10}$ M), norgestrel and gestodene (at $10^{-6}$ M each) are capable of stimulating CAT activity above control levels, while MPA is unable to do so. The addition of the antioestrogen ICI 164, 384 dramatically inhibited the ability of oestradiol, norgestrel and gestodene to stimulate CAT activity (Figure 6).

*Oestradiol, norgestrel and gestodene influence on pS2ERE-CAT activity*

Our next task was to examine whether the interaction was specific to the ERE palindromic sequence, or whether a nonspecific interaction within the plasmid was the cause of our results. In order to determine if the interaction with the ERE was specific, MCF-7 cells were transfected with pS2ERE-CAT, the nonperfect palindrome ERE-CAT plasmid construct (Figure 5). This ERE has been shown to have lower affinity for the ER as compared to vitERE. Oestradiol, norgestrel and gestodene could only weakly stimulate CAT activity at concentrations that could stimulate strong CAT activity with the vitERE (Figure 7).

**Figure 7** The influence of oestradiol, gestodene, and norgestrel on pS2ERE-CAT activity. The protocol used is described in Figure 5, with the exception that the pS2ERE-CAT plasmid was used rather than the vitERE-CAT. The control level represents pS2ERE-CAT activity in the absence of steroid. The open bars represent $10^{-10}$ M oestradiol, $10^{-6}$ M gestodene, $10^{-6}$ M norgestrel, respectively. The hatched bars represent these compounds (at the concentrations indicated) in the presence of ICI 164,384 at $10^{-6}$ M. The influence of MPA on CAT expression was weaker than all other compounds tested (data not shown).

*The ability of norgestrel, gestodene and oestradiol to regulate the expression of progesterone receptor message*

Progesterone receptor expression is known to be increased by oestradiol in MCF-7 cells (Ree *et al.*, 1989). The level of this protein can thus be used as a marker for successful oestrogen receptor activation. We examined the influence of norgestrel and gestodene on progesterone receptor levels (Figure 7). At $10^{-8}$ M, neither gestodene nor norgestrel were able to stimulate production of progesterone receptor protein. However, at $10^{-6}$ M, both compound could stimulate progesterone receptor production. MPA could not stimulate progesterone receptor levels at either of these concentrations.

**Figure 6** The influence of oestradiol, norgestrel, gestodene and MPA on vit ERE-CAT activity with and without the presence of an antioestrogen. MCF-7 cells were transfected with the vitERE-CAT plasmid, and then the cells were treated with oestradiol, norgestrel, gestodene or MPA at the concentrations indicated. The ability of the cell lysate to convert tritiated chloramphenicol to acetylated chloramphenicol was measured by chromatography and scintillation counting. The control lane represents vitERE-CAT activity in the absence of steroid. The open bars indicate the various compounds (oestradiol at $10^{-10}$ M, gestodene at $10^{-6}$ M, norgestrel at $10^{-6}$ M and MPA at $10^{-6}$ M) alone, while the hatched bars indicate these compounds (at the concentrations indicated) in the presence of ICI 164,384 at $10^{-6}$ M.

*PRE-CAT activity by oestradiol, norgestrel, gestodene and MPA with and without the antiprogestin RU486*

Our results to this point suggested that the progestins acted as oestrogens in our model system. It was therefore important to show that the progestins could also interact with the progesterone receptor and induce transcription of genes located near a PRE (Figure 5). However, progestin interaction with the progesterone receptor down-regulates receptor production (VuHai *et al.*, 1978), and at lower concentrations, the progestins did not provide an oestrogen-like stimulation of the progesterone receptor. As a result oestrogen was added to the medium to boost progesterone receptor production. We then transiently transfected a PRE15-CAT plasmid contruct into MCF-7 cells. Results shown on Figure 8 indicate that norgestrel and gestodene are capable of stimulating CAT activity at a lower concentration than the concentration capable of stimulating cell proliferation. MPA could stimulate PRE15-CAT activity despite an inability to stimulate cell proliferation (data not shown). The PRE15-CAT stimulation by norgestrel and gestodene could be blocked by the antiprogestin RU486, whereas RU486 could not block MCF-7 cell proliferation in the growth assays. Incidentally, without oestradiol boost, norgestrel and gestodene could still stimulate PRE-CAT expression in this system at $10^{-6}$ M (a concentration sufficiently oestrogenic to boost progesterone receptor (Table I)), while MPA could not (data not shown).

### Discussion

Our results demonstrate that the 19-nortestosterone derivatives norgestrel and gestodene stimulate MCF-7 cell

**Table I** Progesterone receptor enzyme immunoassay determination of progesterone receptor regulation in MCF-7 cells by oestradiol, norgestrel, gestodene and MPA. The values given are fmol $mg^{-1}$ protein and are done in duplicate

| *Compounds* | *Progesterone receptor content (fmol $mg^{-1}$ protein)* |
|---|---|
| Control | 4.21 +/− 0.74 |
| Oestradiol (10−11 M) | 128.10 +/− 10.93 |
| Oestradiol (10−10 M) | 323.36 +/− 54.56 |
| Norgestrel (10−8 M) | 2.82 +/− 0.00 |
| Norgestral (10−6 M) | 26.24 +/− 2.75 |
| Gestodene (10−8 M) | 3.22 +/− 0.22 |
| Gestodene (10−6 M) | 82.04 +/− 2.4 |
| MPA (10−8 M) | 3.56 +/− 1.44 |
| MPA (10−6 M) | 3.73 +/− 0.35 |

proliferation by activating the oestrogen receptor. Norgestrel and gestodene were capable of stimulating MCF-7 cell proliferation *in vitro*, although they were both less potent and less efficaceous when compared to oestradiol. The stimulation could be blocked by the antioestrogen ICI 164,384 but not by the antiprogestin RU486. We showed that these progestins could activate CAT transcription specifically at the vitellogenin oestrogen responsive element (vit ERE), and this interaction could be blocked by ICI 164,384. We further showed that norgestrel and gestodene could increase progesterone receptor levels, a common marker for oestrogen receptor-mediated transcription.

The progestins norgestrel and gestodene have previously been shown to act differentially in cultured human breast cancer (Iqbal *et al.*, 1986). Despite minor structural differences between the two compounds, the authors had found that gestodene could bind to receptors specific to malignant breast tissue while norgestrel could not. Such binding resulted in growth inhibition. It is surprising that an endogenous receptor should have such high substrate specificity among synthetic progestins. Our results suggest that gestodene and norgestrel act similarly, as would be expected from their structures.

The 19-norprogestins norgestrel and gestodene are capable of stimulating MCF-7 cell growth via interaction with the oestrogen receptor. Oestrogenic potential of progestins is further supported by the recent work of Markiewicz *et al.* (1992). This stimulation occurs in a concentration dependent fashion, and can be inhibited by the pure antioestrogen ICI 164, 384. However, the finding that the antiprogestin RU486 is incapable of inhibiting progestin stimulated growth suggests that the growth stimulatory interaction is not mediated via the progestin receptor. This conclusion is supported by the inability of MPA to stimulate growth (Figure 1) or to stimulate vitERE-CAT activity (Figure 5) while the capable of stimulating PRE15-CAT activity as much as 150% (data not shown).

Although norgestrel and gestodene are capable of stimulating PRE15-CAT activity at very low concentrations (Figure 8), the progestational activity of these compounds do not appear to provide an attenuating effect of their oestrogen-like activity. The MCF-7 cell line proliferates in the presence of the progestin despite the presence of functional PR. We have also tested various 19-norprogestins in the T-47D cell line and have found similar stimulatory activity (unpublished observations). Based upon the correlation between the concentration of progestin required for vitERE-CAT activity and growth stimulation, it is likely that the growth stimulation is mediated via the oestrogen receptor rather than via the progesterone receptor.

The ability of norgestrel to provide effective birth control without the presence of an oestrogen (for example, the minipill and NORPLANT® formulations) had previously been inconsistent with our understanding of progestin biochemistry. If the progestin only acted through the progesterone receptor, the receptor should be downregulated and eventually result in the ineffectiveness of norgestrel. We suggest that norgestrel is also capable of interacting with the

**Figure 8** The influence of gestodene and norgestrel on PRE15-CAT activity with and without the presence of an antiprogestin. Column 1 represents media with $10^{-10}$ M oestradiol (see results section for explanation). All other lanes have $10^{-10}$ M oestradiol in combination with the steroid given. The open bars represent the steroid alone, while the hatched bars represent the steroid combined with $10^{-7}$ M RU486. The transfection procedure was similar to that described in the caption for Figure 5, except that the PRE15-CAT plasmid construct was used and the progesterone receptor was boosted by adding oestradiol to the media.

oestrogen receptor, thus resulting in stimulation of progesterone receptor synthesis and maintained progestational activity.

It is noteworthy that the 19-norprogestins are capable of stimulating MCF-7 cell growth at concentrations as low as $10^{-8}$ M or 30 ng $ml^{-1}$. Such concentrations are similar to plasma progestin concentrations seen in women who use oral contraceptives (Orme *et al.*, 1983; Goldzieher, 1989). Plasma progestin concentrations seen in NORPLANT® users (0.5 nl $ml^{-1}$ or approximately $10^{-10}$ M) are low enough to provide no growth stimulus above control in our model system (Croxatto *et al.*, 1988; Shoupe & Mishell, 1989, Shoupe *et al.*, 1991). Therefore, NORPLANT® may offer advantages over the oral contraceptives by being effective at concentrations below those that stimulate breast cancer cell proliferation.

We suggest that the lowest effective concentration of 19-norprogestins should be pursued. Although developing lower dose formulations has been a priority for hormonal contraceptive manufacturers, our results indicate that the present progestin concentrations are still capable of stimulating human breast cancer. Furthermore, the assumption that progestin-only formulations are devoid of oestrogenic activity appears to be incorrect. For the present time, oral contraceptives should be measured by their total oestrogenicity (i.e. the oestrogen activity of oestrogen plus the oestrogenic activity of the progestin) in order to provide the most prudent and protective means of contraception. This oestrogenicity may also be determined for various formulations used in epidemiological studies that compare oral contraceptive use and breast cancer incidence, and may be one of the distinguishing characteristics betweens the conflicting results of these studies.

This research was sponsored by National Medical Fellowships, Inc. and National Institutes of Health grant RO1-32713-10 for cancer research.

### References

ABRAMS, J.S., PARNES, H. & AISNER, J. (1990). Current status of high-dose progestins in breast cancer. *Sem. Oncol.*, **17**, 68–72.

ALEXANDER, I.E., SHINE, J. & SUTHERLAND, R.L. (1990). Progestin regulation of estrogen receptor messenger RNA in human breast cancer cells. *Mol. Endocinol.*, **4**, 821–828.

ANDERSON, T.J., BATTERSBY, S., KING, R.J.B., MCPHERSON, K. & GOING, J.J. (1989). Oral contraceptive use influences resting breast proliferation. *Hum. Path.*, **20**, 1139–1144.

BERGKVIST, L., ADAMI, H., PERSSON, I., HOOVER, R. & SCHAIRER, C. (1989). The risk of breast cancer after estrogen and estrogen-progestin replacement. *N. Engl. J. Med.*, **321**, 293–297.

BRAUNSBERG, H., COLDHAM, N.G. & WONG, W. (1986). Hormonal therapies for breast cancer; can progestins stimulate growth? *Cancer Letters*, **30**, 213–218.

BUEHRING, G.C. (1988). Oral contraceptives and breast cancer: what has 20 years of research shown? *Biomed. Pharmicother.*, **42**, 525–530.

CASAGRANDE, J.T., LOUIE, E.W., PIKE, M.C., ROY, S., ROSS, R.K. & HENDERSON, B.E. (1979). Incessant ovulation and ovarian cancer. *Lancet*, **2**, 170–173.

COLLETTA, A.A., WAKEFIELD, L.M., HOWELL, F.V., DANIELPOUR, D., BAUM, M. & SPORN, M.B. (1991). The growth inhibition of human breast cancer cells by a novel synthetic progestin involves the induction of transforming growth factor beta. *J. Clin. Invest.*, **87**, 277–283.

CORMIER, E.M. & JORDAN, V.C. (1989). Contrasting ability of antioestrogens to inhibit MCF-7 growth stimulated by estradiol or epidermal growth factor. *Eur. J. Cancer*, **25**, 57–63.

CROXATTO, H.B., DIAZ, S., PAVEZ, M. & BRANDEIS, A. (1988). Estradiol plasma levels during long-term treatment with NORPLANT® subdermal implants. *Contraception*, **38**, 465–475.

DAUVOIS, S., SIMARD, J., DUMONT, M., HAAGENSEN, D.E. & LABRIE, F. (1990). Opposite effects of estrogen and the progestin R5020 on cell proliferation and GCDFP-15 expression in ZR-75-1 human breast cancer cells. *Mol. Cell. Endocrinol.*, **73**, 171–178.

DRILL, V.A. (1977). History of the first oral contraceptive. *J. Tox. Env. Health*, **3**, 133–138.

GOLDZIEHER, J.W. (1989). Pharmacology of contraceptive steroids: a brief review. *Am. J. Obstet. Gynecol.*, **5**, 1260–1264.

HASLAM, S.Z. (1988). Progesterone effects on deoxyribonucleic acid synthesis in normal mouse mammary glands. *Endocrinology*, **122**, 464–470.

HENDERSON, B.E., ROSS, R.K., LOBO, R.A., PIKE, M.C. & MACK, T.M. (1988). Re-evaluating the role of progestogen therapy after the menopause. *Fert. Steril.*, **49**, 9s–15s.

HULKA, B.S., CHAMBLESS, L.E., KAUFMAN, D.G., FOWLER, W.C. & GREENBERG, B.G. (1982). Protection against endometrial carcinoma by combination-product oral contraceptives. *J. Am. Med. Assoc.*, **247**, 475–477.

IQBAL, M.J., COLLETTA, A.A., HOUMAYOUN-VALYANI, S.D. & BAUM, M. (1986). Differences in oestrogen receptors in malignant and normal breast tissue as identified by the binding of a new synthetic progestogen. *Br. J. Cancer*, **54**, 447–452.

JENG, M.-H. & JORDAN, V.C. (1991). Growth stimulation and differential regulation of transforming growth factor beta 1 (TGF beta 1), TGF beta 2, and TGF beta 3 messenger RNA levels by norethindrone in MCF-7 human breast cancer cells. *Mol. Endocrinol.*, **5**, 1120–1128.

KAUFMAN, D.W., SHAPIRO, T., SLONE, D., ROSENBERG, L., MIETTINEN, O.S., STOLLEY, P.D., KNAPP, R.C., LEAVITT, T., WATRING, W.G., ROSENSHEIN, N.B., LEWIS, J.L., SCHOTTENFELD, D. & ENGLE, R.L. (1980). Decreased risk of endometrial cancer among oral contraceptive users. *N. Engl. J. Med.*, **303**, 1045–1047.

KAY, C.R. & HANNAFORD, P.C. (1988). Breast cancer and the pill-A further report from the Royal College of General Practitioners' oral contraceptive study. *Br. J. Cancer*, **58**, 675–680.

KLOCK, G., STRAHLE, U. & SCHUTZ, G. (1987). Oestrogen and glucocorticoid responsive elements are closely related but distinct. *Nature*, **329**, 734–736.

LABARCA, C. & PAIGEN, K. (1980). A simple, rapid, and sensitive DNA assay procedure. *Anal. Biochem*, **102**, 344–352.

LAYDE, P.M. (1982). Long-term oral contraceptive use and the risk of cancer. Read before the First Annual Conference on Family Planning, Atlanta, Oct 4.

LIANG, A.P., LEVENSON, A.G., LAYDE, P.M., SHELTON, J.D., HATCHER, R.A., POTTS, M. & MICHELSON, M.J. (1983). Risk of breast, uterine corpus, and ovarian cancer in women receiving medroxyprogesterone injections. *JAMA*, **249**, 2909–2912.

LØBER, J., ROSE, C., SALIMTSCHIK, M. & MOURIDSEN, H.T. (1981). Treatment of advanced breast cancer with progestins. *Acta. Obstet. Gynecol. Scand. Suppl.*, **101**, 39–46.

LONGMAN, S.M. & BUEHRING, G.C. (1987). Oral contraceptives and breast cancer *in vitro* effect of contraceptive steroids on human mannary cell growth. *Cancer*, **59**, 281–287.

MACGREGOR, G. & CASKEY, C.T. (1989). Construction of plasmids that express *E. coli* beta-galactosidase in mammalian cells. *Nucleic Acid Res.*, **17**, 2365.

MARKIEWICZ, L., HOCHBERG, R.B. & GURPIDE, E. (1992). Intrinsic estrogenicity of some progestagenic drugs. *J. Steroid Biochem. Molec. Biol.*, **41**, 53–58.

MCGOWAN, L., PARENT, L., LEDNAR, W. & NORRIS, H.J. (1979). The woman at risk for developing ovarian cancer. *Gynecol. Oncol.*, **7**, 325.

MCPHERSON, K., NEIL, A., VESSEY, M.P. & DOLL, R. (1983). Oral contraceptives and breast cancer. *Lancet*, **2**, 1414–1415.

MCPHERSON, K., VESSEY, M.P., NEIL, A., DOLL, R., JONES, L. & ROBERTS, M. (1987). Early oral contraceptive use and breast cancer: results of another case-control study. *Br. J. Cancer*, **56**, 653–660.

MEIRIK, O., LUND, E., ADAMI, H., BERGSTROM, R., CHRISTOFFERSON, T. & BERGSJOO, P. (1986). Oral contraceptive use and breast cancer in young women. *Lancet*, **2**, 650–654.

NEWHOUSE, M.L., PEARSON, R.M. FULLERTON, J.M., BOESEN, E.A. & SHANNON, H.S. (1977). A case control study of carcinoma of the ovary. *Br. J. Prev. Soc. Med.*, **31**, 148–153.

OLSSON, H., LANDIN-OLSSON, M., MÖLLER, T.R., RANSTAM, J. & HOLM, P. (1985). Oral contraceptive use and breast cancer in young women in Sweden. *Lancet*, **2**, 748–749.

OLSSON, H., MÖLLER, T.R. & RANSTAM, J. (1989). Early oral contraceptive use and breast cancer among premenopausal women: final report from a study in southern Sweden. *J. Natl Cancer Inst.*, **81**, 1000–1004.

ORME, M.L.E., BACK, D.J. & BRECKENRIDGE, A.M. (1983). Clinical pharmacokinetics of oral contraceptive steroids. *Clin. Pharmacokin.*, **8**, 95–136.

PAPA, V., HARTMANN, K.K.P., ROSENTHAL, S.M., MADDUX, B.A., SIITERI, P.K. & GOLDFINE, I.D. (1991). Progestins induce down-regulation on insulin-like growth factor-I (IGF-I) receptors in human breast cancer cells: potential autocrine role of IGF-II. *Mol. Endocrinol.*, **5**, 709–717.

PAUL, C., SKEGG, D.C.G. & SPEARS, G.F.S. (1989). Depot medroxyprogesterone (Depo-Provera) and risk of breast cancer. *Br. Med. J.*, **299**, 759–762.

PETO, J. (1989). Oral contraceptives and breast cancer: is the CASH study really negative? *Lancet*, **2**, 552.

PHILLIPS, A. (1990). The selectivity of a new progestin. *Acta. Obstet. Gynecol. Scand. Suppl.*, **152**, 21–24.

PIKE, M.C., HENDERSON, B.E., KRAILO. M.D., DUKE, A. & ROY, S. (1983). Breast cancer in young women and use of oral contraceptives: possible modifying effect of formulation and age at use. *Lancet*, **2**, 926–929.

POLLOW, K., JUCHEM, M., GRILL, H.-J., ELGER, W., BEIER, S., SCHMIDT-GOLLWITZER, K. & MANZ, B. (1989). Gestodene: a novel synthetic progestin–characterization of binding to receptor and serum proteins. *Contraception*, **40**, 325–341.

REE, A.H., LANDMARK, W., ESKILD, F., LEVY, F.O., LAHOOTI, H., JAHNSEN, T., AAKRAAG, A. & HANSSON, V. (1989). Autologous down-regulation of messenger ribonucleic acid and protein levels for estrogen receptors in MCF-7 cells: an inverse correlation to progesterone receptor levels. *Endocrinology*, **124**, 2577–2583.

ROSENBERG, L., SHAPIRO, S., SLONE, D., KAUFMAN, D.W., HELMRICH, S.P., MIETTINEN, O.S., STOLLEY, P.D., ROSENSHEIN, N.B., SCHOTTENFELD, D. & ENGLE, R.L. (1982). Epithelial ovarian cancer and combination oral contraceptives. *J. Am. Med. Assoc.*, **247**, 3210–3212.

SALMI, T. (1979). Risk factors in endometrial carcinoma with special reference to the use of estrogens. *Acta. Obstet. Gynecol. Scand.*, **86**, 1–119.

SATTIN, R.W., RUBIN, G.L., WINGO, P.A., WEBSTER, L.A. & ORY, H.W. (1986). Oral-contraceptive use and the risk of breast cancer. *N. Engl. J. Med.*, **315**, 405–411.

SCHLESSELMAN, J.J. (1989). Cancer of the breast and reproductive tract in relation to use of oral contraceptives. *Contraception*, **40**, 1–38.

SHOUPE, D. & MISHELL, D.R. (1989). Norplant: subdermal implant system for long-term contraception. *Am. J. Obstet. Gynecol.*, **160**, 1286–1292.

SHOUPE, D., MISHELL, D.R., BOPP, B.L. & FIELDING, M. (1991). The significance of bleeding patterns in norplant implant users. *Obstet. Gynecol.*, **77**, 256-260.

SOULE, H.D., VAZQUEZ, J., LONG, A., ALBERT, S. & BRENNAN, M. (1973). A human cell line from a pleural effusion derived from a breast carincoma. *J. Natl Cancer Inst.*, **51**, 1409-1416.

STADEL, B.V. (1988). Oral contraceptives and premenopausal breast cancer in nulliparous women. *Contraception*, **38**, 287-299.

STOLL, B.A. (1967). Progestin therapy of breast cancer: comparison of agents. *Br. Med. J.*, **3**, 338-341.

SUTHERLAND, R.L., HALL, R.E., PANG, G.Y.N., MUSGROVE, E.A. & CLARKE, C.L. (1988). Effect of medroxyprogesterone acetate on proliferation and cell cycle kinetics of human mammary carcinoma cells. *Cancer Res.*, **48**, 5084-5091.

VUHAI, M.T., WAREMBOURG, M. & MILGROM, E. (1977). Hormonal control of progesterone receptors. *Ann. N.Y. Acad. Sci.*, **286**, 199-209.

WAKELING, A.E. & BOWLER, J. (1988). Biology and mode of action of pure antiestrogens. *J. Steroid Biochem.*, **30**, 141-147.

WEISS, N.S. & SAYVETS, T. (1980). Incidence of endometrial cancer in relation to the use of oral contraceptives. *N. Engl. J. Med.*, **302**, 551-554.

WISEMAN, R.A. (1983). Oral contraceptives and breast cancer rates. *Lancet*, **2**, 1415-1416.